# A Simplified Quantitative Real-Time PCR Assay for Monitoring SARS-CoV-2 Growth in Cell Culture

**DOI:** 10.1128/mSphere.00658-20

**Published:** 2020-09-02

**Authors:** Christian Shema Mugisha, Hung R. Vuong, Maritza Puray-Chavez, Adam L. Bailey, Julie M. Fox, Rita E. Chen, Alex W. Wessel, Jason M. Scott, Houda H. Harastani, Adrianus C. M. Boon, Haina Shin, Sebla B. Kutluay

**Affiliations:** a Department of Molecular Microbiology, Washington University School of Medicine, St. Louis, Missouri, USA; b Department of Pathology & Immunology, Washington University School of Medicine, St. Louis, Missouri, USA; c Department of Medicine, Washington University School of Medicine, St. Louis, Missouri, USA; University of Texas Southwestern Medical Center

**Keywords:** SARS-CoV-2, antivirals, assay development, viral entry, virus replication

## Abstract

Severe acute respiratory syndrome coronavirus 2 (SARS-CoV-2) has infected millions within just a few months, causing severe respiratory disease and mortality. Assays to monitor SARS-CoV-2 growth *in vitro* depend on time-consuming and costly RNA extraction steps, hampering progress in basic research and drug development efforts. Here, we developed a simplified quantitative real-time PCR assay that bypasses viral RNA extraction steps and can monitor SARS-CoV-2 growth from a small amount of cell culture supernatants. In addition, we show that this approach is easily adaptable to numerous other RNA and DNA viruses. Using this assay, we screened the activities of a number of compounds that were predicted to alter SARS-CoV-2 entry and replication as well as HIV-1-specific drugs in a proof-of-concept study. We found that E64D (inhibitor of endosomal proteases cathepsin B and L) and apilimod (endosomal trafficking inhibitor) potently decreased the amount of SARS-CoV-2 RNA in cell culture supernatants with minimal cytotoxicity. Surprisingly, we found that the macropinocytosis inhibitor ethylisopropylamiloride (EIPA) similarly decreased SARS-CoV-2 RNA levels in supernatants, suggesting that entry may additionally be mediated by an alternative pathway. HIV-1-specific inhibitors nevirapine (a nonnucleoside reverse transcriptase inhibitor [NNRTI]), amprenavir (a protease inhibitor), and allosteric integrase inhibitor 2 (ALLINI-2) modestly inhibited SARS-CoV-2 replication, albeit the 50% inhibitory concentration (IC_50_) values were much higher than that required for HIV-1. Taking the data together, this simplified assay will expedite basic SARS-CoV-2 research, be amenable to mid-throughput screening assays (i.e., drug, CRISPR, small interfering RNA [siRNA], etc.), and be applicable to a broad number of RNA and DNA viruses.

**IMPORTANCE** Severe acute respiratory syndrome coronavirus 2 (SARS-CoV-2), the etiological agent of the coronavirus disease 2019 (COVID-19) pandemic, is continuing to cause immense respiratory disease and social and economic disruptions. Conventional assays that monitor SARS-CoV-2 growth in cell culture rely on costly and time-consuming RNA extraction procedures, hampering progress in basic SARS-CoV-2 research and development of effective therapeutics. Here, we developed a simple quantitative real-time PCR assay to monitor SARS-CoV-2 growth in cell culture supernatants that does not necessitate RNA extraction and that is as accurate and sensitive as existing methods. In a proof-of-concept screen, we found that E64D, apilimod, EIPA, and remdesivir can substantially impede SARS-Cov-2 replication, providing novel insight into viral entry and replication mechanisms. In addition, we show that this approach is easily adaptable to numerous other RNA and DNA viruses. This simplified assay will undoubtedly expedite basic SARS-CoV-2 and virology research and be amenable to use in drug screening platforms to identify therapeutics against SARS-CoV-2.

## Abstract (precis)

Severe acute respiratory syndrome coronavirus 2 (SARS-CoV-2), the etiological agent of the coronavirus disease 2019 (COVID-19) pandemic, is continuing to cause immense respiratory disease and social and economic disruptions. Conventional assays that monitor SARS-CoV-2 growth in cell culture rely on costly and time-consuming RNA extraction procedures, hampering progress in basic SARS-CoV-2 research and development of effective therapeutics. Here, we developed a simple quantitative real-time PCR assay to monitor SARS-CoV-2 growth in cell culture supernatants that does not necessitate RNA extraction and that is as accurate and sensitive as existing methods. In a proof-of-concept screen, we found that E64D, apilimod, EIPA, and remdesivir can substantially impede SARS-Cov-2 replication, providing novel insight into viral entry and replication mechanisms. In addition, we show that this approach is easily adaptable to numerous other RNA and DNA viruses. This simplified assay will undoubtedly expedite basic SARS-CoV-2 and virology research and be amenable to use in drug screening platforms to identify therapeutics against SARS-CoV-2.

## OBSERVATION

Severe acute respiratory syndrome coronavirus 2 (SARS-CoV-2) is continuing to cause substantial morbidity and mortality around the globe (1, 2). Lack of a simple assay to monitor virus growth is slowing progress in basic SARS-CoV-2 research as well as drug discovery. Current methods used to quantify SARS-CoV-2 growth in cell culture supernatants rely on time-consuming and costly RNA extraction protocols followed by quantitative real-time PCR (qRT-PCR) (3). In this study, we developed a simplified qRT-PCR assay that bypasses the RNA extraction steps, can detect viral RNA from as little as 5 μl of cell culture supernatants, and works equally well with TaqMan and SYBR green-based detection methods.

**FIG 1:**
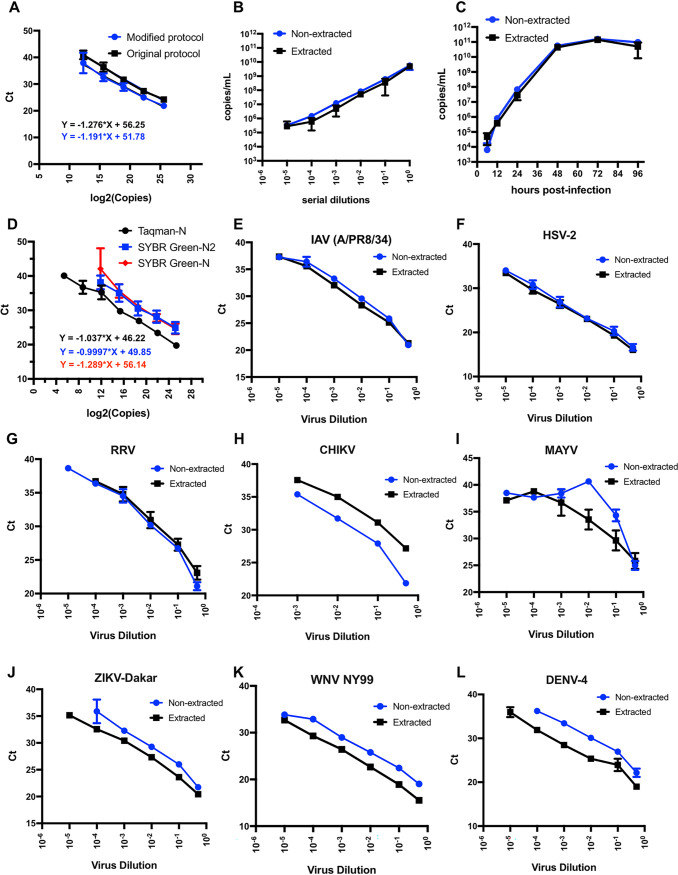
Development of a simplified qRT-PCR assay for SARS-CoV-2 viral RNA detection in cell culture supernatants. (A) Serially diluted RNA standards were either directly subjected to qRT-PCR or processed as described in the modified protocol detailed in the text prior to qRT-PCR. Log_2_ copy numbers are plotted against the cycle threshold (*C_T_*) values. Linear regression analysis was done to obtain the equations. Data show averages of results from three independent biological replicates. Error bars show standard errors of the means (SEM). (B) Comparison of the efficiency and detection ranges for quantifying SARS-CoV-2 RNA using purified RNA or lysed supernatants from virus stocks. Data are derived from three independent replicates. Error bars show the SEM. (C) Vero E6 cells were infected at a multiplicity of infection (MOI) of 0.01, and cell culture supernatants were analyzed for SARS-CoV-2 RNA following the conventional RNA extraction protocol versus the modified protocol developed here at various times postinfection. Cell-associated viral RNA was analyzed in parallel following RNA extraction for reference. Data are from three independent biological replicates. Error bars show the SEM. (D) Illustration of the efficiency and detection ranges of TaqMan-based and SYBR green-based qRT-PCR assays quantifying known amounts of SARS-CoV-2 RNA. Data are from 2 or 3 independent replicates. Error bars show the SEM. (E to L) The indicated viruses were subjected to RNA or DNA extraction (extracted) and diluted 10-fold or used directly following dilution (nonextracted) in the SYBR green-based (E to I) or TaqMan-based (J and L) qRT-PCR assay as described above. Samples were normalized such that equivalent amounts of the original virus stock were added to PCRs for extracted and nonextracted samples. Plots show the corresponding cycle threshold values (Ct, *y* axis) per virus dilution (*x* axis). Data are from two independent replicates, with error bars showing the SEM.

A widely used assay to measure virus growth in the retrovirology field relies on determining the activity of virion-associated reverse transcriptase enzyme collected from a small amount of infected cell culture supernatants (4). We reasoned that we could adapt this approach to monitor SARS-CoV-2 growth. First, we tested whether the more stringent lysis conditions used to inactivate SARS-CoV-2 would interfere with the subsequent qRT-PCR step. To do so, a 5-μl volume of serially diluted RNA standards prepared by *in vitro* transcription from a plasmid containing the entire SARS-CoV-2 nucleoprotein (N) gene was mixed with 5 μl of 2× RNA lysis buffer (2% Triton X-100, 50 mM KCl, 100 mM Tris-HCl [pH 7.4], 40% glycerol, 0.4 U/μl of Superase•IN [Life Technologies]), followed by addition of 90 μl of 1× core buffer [5 mM (NH_4_)_2_SO_4_, 20 mM KCl, 20 mM Tris-HCl (pH 8.3)]. An 8.5-μl volume of the diluted samples was added to 11.5 μl of a reaction mixture consisting of 10 μl of a 2× TaqMan RT-PCR mixture, 0.5 μl of a 40× TaqMan reverse transcription enzyme mixture (containing ArrayScript UP reverse transcriptase, RNase inhibitor), and 1 μl of a mixture containing 10 pmol of forward and reverse primers as well as 2 pmol of TaqMan probe (see Table S1 in the supplemental material), resulting in a final reaction volume of 20 μl. The reactions were run on a ViiA 7 real-time PCR system (Applied Biosystems) using the following cycling parameters: 48°C for 15 min, 95°C for 10 min, and 50 cycles of 95°C for 15 s and 60°C for 1 min of signal acquisition. We found that the modified sample preparations did not impact the sensitivity, efficiency, or dynamic range of the qRT-PCR assay as evident in the virtually identical cycle threshold (*C_T_*) values obtained for a given RNA concentration and the similar slopes of linear regression curves (Fig. 1A).

10.1128/mSphere.00658-20.2 TABLE S1 Sequences of the primers and probes used in this study. Download Table S1, DOCX file, 0.08 MB. Copyright © 2020 Shema Mugisha et al. 2020 Shema Mugisha et al. This content is distributed under the terms of the Creative Commons Attribution 4.0 International license.

To determine whether this approach would work equally well for other virus preparations, 100 μl of virus stock (1.4 × 10^5^ PFU) was lysed via the addition of an equal volume of buffer containing 40 mM Tris-HCl, 300 mM NaCl, 10 mM MgCl_2_, 2% Triton X-100, 2 mM dithiothreitol (DTT), 0.4 U/μl Superase•IN RNase inhibitor, and 0.2% NP-40. RNA was then extracted using a Zymo RNA clean and concentrator-5 kit and was serially diluted afterwards. In parallel, 5 μl of virus stock and corresponding serial dilutions prepared in cell culture media were lysed in 2× RNA lysis buffer and processed as described above. Samples were analyzed by qRT-PCR alongside RNA standards. A standard curve was constructed by plotting the cycle threshold (*C_T_*) value against the corresponding log_2_(copy number) of the RNA standards, which was subsequently used to determine copy numbers in samples. We then calculated the number of copies per milliliter of the original virus stock, assuming 100% recovery for samples subjected to RNA extraction. We found that the modified assay performed as well as if not better than the standard assay, with a similarly broad dynamic range (Fig. 1B).

We next used this assay to monitor virus growth on infected Vero cells. Cell culture supernatants containing virus collected at various times postinfection (pi) were either used to extract viral RNA or subjected to qRT-PCR directly (nonextracted) as described above. The modified assay performed with nonextracted samples yielded virtually identical numbers of copies/ml of SARS-CoV-2 RNA in cell culture supernatants even at low concentrations of viral RNAs (Fig. 1C). Collectively, these results suggest that the step of RNA extraction from cell culture supernatants can be bypassed without any compromise regarding the sensitivity or the dynamic range of qRT-PCR detection.

Next, we wanted to test whether this assay could work as well as SYBR green-based detection methods. In addition to the N primer pair used in the TaqMan-based assays described above, we utilized the N2 primer set designed by CDC that targets the N region of the SARS-CoV-2 genome (Table S1). Serially diluted RNA standards were processed in RNA lysis and core buffers, and 7.5 μl of each dilution was used in a 20-μl SYBR green qRT-PCR reaction mixture containing 10 μl of a 2× PowerUp SYBR green mixture (Life Technologies catalog no. A25742), 1.25 units/μl of MultiScribe reverse transcriptase (Applied Biosystems), 1× random primers, and 25 pmol of forward and reverse primers. Both primer pairs yielded reasonably broad dynamic ranges but were modestly less sensitive than the TaqMan-based assays, with a detection limit of ∼3,500 RNA copies/ml (Fig. 1D).

We next tested whether this simplified qRT-PCR assay can be adapted to detection of other RNA and DNA viruses. Dilutions of stocks of influenza A virus (IAV/PR8), herpes simplex virus 2 (HSV-2), alphaviruses (Ross River virus [RRV], Chikungunya virus [CHIKV], and Mayarovirus [MAYV]), and flaviviruses (dengue virus [DENV-4], West Nile virus [WNV NY99], and Zika virus [ZIKV-Dakar]) collected from cell culture supernatants were subjected to either RNA/DNA extraction or the simplified lysis protocol as described above followed by SYBR green-based or TaqMan-based qRT-PCR with the indicated primers (Table S1). For HSV-2, the reaction mixture did not include the reverse transcription enzyme and the initial reverse transcription step was skipped. We found that for IAV (Fig. 1E), HSV-2 (Fig. 1F), and RRV (Fig. 1G), the nonextracted samples worked equally well and that the nonextracted samples gave lower *C_T_* values for CHIKV across various virus dilutions (Fig. 1H). For MAYV, the dynamic range obtained from nonextracted samples was low compared to that obtained from extracted samples (Fig. 1I), likely due to the incompatibility between the lysis and PCR conditions. Although the *C_T_* values were generally higher for the nonextracted samples of ZIKV (Fig. 1J), WNV (Fig. 1K), and DENV (Fig. 1L), the dynamic range was still broad, with similar PCR efficiencies seen in the comparisons between extracted and nonextracted samples. Taken together, these results demonstrate that the simplified qRT-PCR developed here can in principle be easily adapted to a large number of viruses provided that the lysis conditions are appropriate and working primer sets are present.

**FIG 2:**
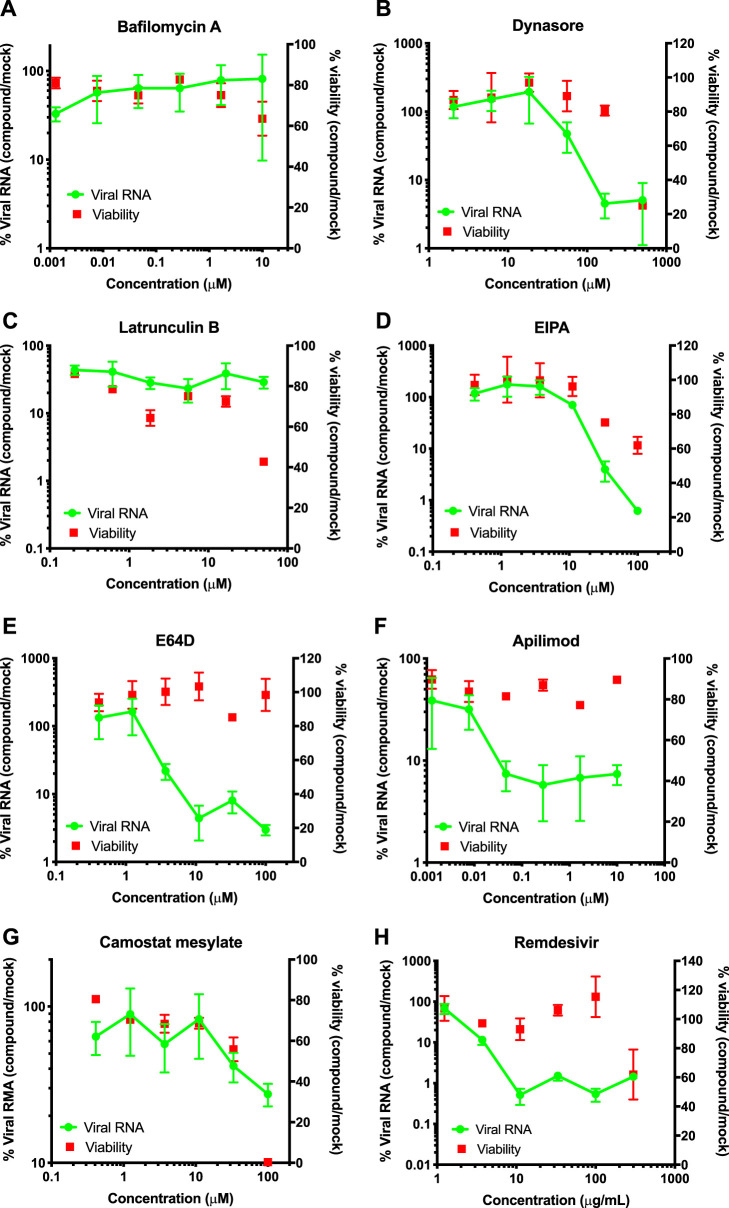
A compound screen to validate SARS-CoV-2-specific inhibitors and entry pathways. Vero E6 cells were infected with SARS-CoV-2 at an MOI of 0.01, and inhibitors were added concomitantly at the concentrations shown in the figures following virus adsorption. Supernatants from infected cells were lysed and used in a SYBR green-based qRT-PCR to quantify the viral RNA in cell culture supernatants. Compound cytotoxicity was monitored by the use of a RealTime-Glo MT cell viability assay kit (Promega) in parallel plates. Data show the cumulative results from 2 to 5 independent biological replicates. Error bars show the SEM. (A) Bafilomycin A. (B) Dynasore. (C) Latrunculin B. (D) EIPA. (E) E64D. (F) Apilimod. (G) Camostat mesylate. (H) Remdesivir.

One immediate application of this simplified assay is in mid-throughput drug screening platforms (i.e., compound, CRISPR, and small interfering RNA [siRNA] screens) given the ease of quantitatively assessing viral growth from small quantities of cell culture media containing virions. To demonstrate this, we next conducted a proof-of-concept drug screen to validate the antiviral activities of various compounds that have been reported to inhibit SARS-CoV-2 and HIV-1 replication as well as nonspecific entry inhibitors (Table S2). Vero E6 cells plated in 96-well plates were infected in the presence of various concentrations of the indicated compounds. Viral RNA in cell culture supernatants was quantified by the SYBR green-based qRT-PCR assay as described above at 6, 24, and 48 h postinfection (hpi). Compound cytotoxicity was assessed in parallel by the use of a RealTime-Glo MT cell viability assay (Promega). While viral RNA was at background levels at 6 hpi (data not shown), we found that, at 24 hpi, remdesivir (inhibitor of RNA-dependent RNA polymerase [5]), E64D (inhibitor of the endosomal protease cathepsin B, K, and L), and apilimod (PIKfyve inhibitor resulting in endosomal trafficking defects [6, 7]) substantially decreased SARS-CoV-2 viral RNAs in supernatants (Fig. 2). IC_50_ (50% inhibitory concentration) values of these compounds (2.8 μg/ml remdesivir, 3.3 μM E64D, and 12 nM apilimod) were within the same range as the IC_50_ values published for these compounds (6–8) (Fig. 2). Similar results were obtained at 48 hpi, albeit E64D and apilimod appeared to be less potent at this time point due to either virus overgrowth or compound turnover (data not shown). We found that ethylisopropylamiloride (EIPA), which inhibits Na^+^/H^+^ exchanger and macropinocytosis, substantially decreased viral RNA in supernatants at subcytotoxic levels (Fig. 2D), suggesting that macropinocytosis may contribute to viral entry and/or subsequent steps in virus replication. HIV-1-specific inhibitors nevirapine, amprenavir, and allosteric integrase inhibitor 2 (ALLINI-2) modestly inhibited SARS-CoV-2 replication (without apparent cytotoxicity) at high concentrations, albeit the concentrations required for this inhibition were much higher than those that inhibit HIV-1 (see Fig. S1 in the supplemental material). Overall, these findings demonstrate that this simplified assay can be adapted for screening platforms and support previous reports which demonstrated that SARS-CoV-2 entry is dependent on processing of the Spike protein by cellular proteases and requires endosomal fusion (7, 9, 10).

10.1128/mSphere.00658-20.1 FIG S1 A screen to test the antiviral activities of various HIV-1-specific inhibitors. Vero E6 cells were infected with SARS-CoV-2 at a multiplicity of infection (MOI) of 0.01, and inhibitors were added concomitantly at the concentrations shown in the figures following virus adsorption. Supernatants from infected cells were lysed and used in a SYBR green-based Q-RT PCR assay to quantify the viral RNA in cell culture supernatants. Compound cytotoxicity was monitored by the use of a RealTime-Glo MT cell viability assay kit (Promega) in parallel plates. Data show the cumulative results from 2 or 3 independent biological replicates. Error bars show standard errors of the means (SEM). Download FIG S1, TIF file, 1.3 MB. Copyright © 2020 Shema Mugisha et al. 2020 Shema Mugisha et al. This content is distributed under the terms of the Creative Commons Attribution 4.0 International license.

10.1128/mSphere.00658-20.3 TABLE S2 Sources and properties of the compounds used in this study. Download Table S2, DOCX file, 0.2 MB. Copyright © 2020 Shema Mugisha et al. 2020 Shema Mugisha et al. This content is distributed under the terms of the Creative Commons Attribution 4.0 International license.

In conclusion, we have developed a simple qRT-PCR assay to monitor the growth of SARS-CoV-2 as well as other viruses from cell culture supernatants, bypassing the time-consuming and costly RNA extraction procedures. This simplified assay will undoubtedly expedite basic SARS-CoV-2 research, might be amenable to use in mid-throughput screens to identify chemical inhibitors of SARS-CoV-2, and can be applicable to the study of numerous other RNA and DNA viruses.
