# Supplementary material for: A Simplified Quantitative Real-Time PCR Assay for Monitoring SARS-CoV-2 Growth in Cell Culture
Source: mSphere. 2020 Sep 2;5(5):e00658-20. doi: 10.1128/mSphere.00658-20 (PMC7471006; doi:10.1128/mSphere.00658-20)
Supplement: TABLE S1 [file mSphere.00658-20-st001.docx]

| Virus (Gene) | Primer/Probe Sequence (Forward/Reverse) |
| --- | --- |
| SARS-CoV-2 (N) | 5’-ATGCTGCAATCGTGCTACAA-3’ (F) |
| SARS-CoV-2 (N) | 5’-GACTGCCGCCTCTGCTC-3’ (R) |
| SARS-Cov-2 (N) | 5’-FAM/TCAAGGAAC/ZEN/AACATTGCCAA/3IABkFQ/ |
| SARS-CoV-2 (N2) | 5’-TTACAAACATTGGCCGCAAA-3’ (F) |
| SARS-CoV-2 (N2) | 5’-GCGCGACATTCCGAAGAA-3’ (R) |
| IAV A/PR8/34 (M) | 5’-AAGACCAATCCTGTCACCTCTGA-3’ (F) |
| IAV A/PR8/34 (M) | 5’-CAAAGCGTCTACGCTGCAGTCC-3’ (R) |
| HSV-2 (ICP27) | 5’-TGT CGG AGA TCG ACT ACA CG-3’ (F) |
| HSV-2 (ICP27) | 5’-CGGTGCGTGTCCAGTATTTC-3’ (R) |
| CHIKV (E1) | 5’-TCGACGCGCCCTCTTTAA-3’ (F) |
| CHIKV (E1) | 5’-ATCGAATGCACCGCACAC T-3’ (R) |
| RRV (nsP3) | 5’-GTGTTCTCCGGAGGTAAAGATAG-3’ (F) |
| RRV (nsP3) | 5’-TCGCGGCAATAGATGACTAC-3’ (R) |
| MAYV (nsp1-3) | 5’-AAGCTCTTCCTCTGCATTGC-3’ (F) |
| MAYV (nsp1-3) | 5’-TGCTGGAAACGCTCTCTGTA-3’ (R) |
| ZIKV-Dakar (pp) | 5’-TTCGGACAGCCGTTGTCCAACACAAG-3’ (F) |
| ZIKV-Dakar (pp) | 5’-CCACCAATGTTCTCTTGCAGACATATTG-3’ (R) |
| ZIKV-Dakar (pp) | 5’-FAM/AGCCTACCT/ZEN/TGACAAGCAGTC/3IABkFQ/ |
| WNV-NY99 (pp) | 5’-TCAGCGATCTCTCCACCAAAG-3’ (F) |
| WNV-NY99 (pp) | 5’-GGGTCAGCACGTTTGTCATTG-3’ (R) |
| WNV-NY99 (pp) | 5’-FAM/TGCCCGACC/ZEN/ATGGGAGAAGCTC/3IABkFQ/ |
