# Supplementary figures and images for: A Simplified Quantitative Real-Time PCR Assay for Monitoring SARS-CoV-2 Growth in Cell Culture
Source: mSphere. 2020 Sep 2;5(5):e00658-20. doi: 10.1128/mSphere.00658-20 (PMC7471006; doi:10.1128/mSphere.00658-20)

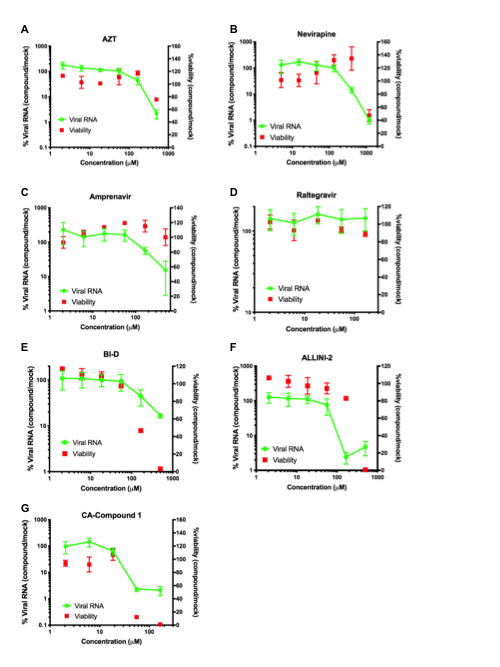

Supplement: FIG S1 [file mSphere.00658-20-sf001.tif]
