# Supplementary material for: A Simplified Quantitative Real-Time PCR Assay for Monitoring SARS-CoV-2 Growth in Cell Culture
Source: mSphere. 2020 Sep 2;5(5):e00658-20. doi: 10.1128/mSphere.00658-20 (PMC7471006; doi:10.1128/mSphere.00658-20)
Supplement: TABLE S2 [file mSphere.00658-20-st002.docx]

| Compound name | Source | Function | Reference |
| --- | --- | --- | --- |
| Camostat mesylate | Tocris Bioscience | TMPRSS2 inhibitor | (1), (2) |
| Bafilomycin A | Sigma Aldrich | Inhibits receptor mediated endocytosis | (3) |
| Apilimod | A gift of Sean Whelan | Inhibits PIKfyve kinase and endosomal trafficking | (3, 4) |
| E64D | Sigma Aldrich | Inhibits cathepsins B and L | (3) |
| EIPA | Sigma Aldrich | Inhibits macropinocytosis | (5) |
| Dynasore | Sigma Aldrich | inhibits dymanin and clathrin-mediated endocytosis | (5) |
| Latrunculin B | Sigma Aldrich | Inhibits actin polymerization |  |
| Remdesivir | A gift of Gaya Amarasinghe | RNA-dependent RNA polymerase | (6) |
| Compound 1 | Custom synthesis | Inhibits HIV-1 capsid stability | (7, 8) |
| Nevirapine | NIH AIDS Reagents | HIV-1 non-nucleoside reverse transcriptase inhibitor (NNRTI) | (9) |
| Azidothymidine (AZT) | NIH AIDS Reagents | HIV-1 nucleoside reverse transcriptase inhibitor (NRTI) | (10, 11) |
| Raltegravir | NIH AIDS Reagents | Integrase strand transfer inhibitor | (12) |
| ALLINI-2 | Custom synthesis | Allosteric integrase inhibitor | (13) |
| BI-D | Custom synthesis | Allosteric integrase inhibitor | (14) |
| Amprenavir | NIH AIDS Reagents | HIV-1 protease inhibitor | (15) |

**REFERENCES**

- 1. **Shang J, Wan Y, Luo C, Ye G, Geng Q, Auerbach A, Li F.** 2020. Cell entry mechanisms of SARS-CoV-2. Proc Natl Acad Sci U S A **117:**11727-11734.
- 2. **Hoffmann M, Kleine-Weber H, Schroeder S, Kruger N, Herrler T, Erichsen S, Schiergens TS, Herrler G, Wu NH, Nitsche A, Muller MA, Drosten C, Pohlmann S.** 2020. SARS-CoV-2 Cell Entry Depends on ACE2 and TMPRSS2 and Is Blocked by a Clinically Proven Protease Inhibitor. Cell **181:**271-280 e278.
- 3. **Ou X, Liu Y, Lei X, Li P, Mi D, Ren L, Guo L, Guo R, Chen T, Hu J, Xiang Z, Mu Z, Chen X, Chen J, Hu K, Jin Q, Wang J, Qian Z.** 2020. Characterization of spike glycoprotein of SARS-CoV-2 on virus entry and its immune cross-reactivity with SARS-CoV. Nat Commun **11:**1620.
- 4. **Kang Y-L, Chou Y-Y, Rothlauf PW, Liu Z, Soh TK, Cureton D, Case JB, Chen RE, Diamond MS, Whelan SPJ, Kirchhausen T.** 2020. Inhibition of PIKfyve kinase prevents infection by EBOV and SARS-CoV-2. bioRxiv doi:10.1101/2020.04.21.053058**:**2020.2004.2021.053058.
- 5. **Burkard C, Verheije MH, Wicht O, van Kasteren SI, van Kuppeveld FJ, Haagmans BL, Pelkmans L, Rottier PJ, Bosch BJ, de Haan CA.** 2014. Coronavirus cell entry occurs through the endo-/lysosomal pathway in a proteolysis-dependent manner. PLoS Pathog **10:**e1004502.
- 6. **Sheahan TP, Sims AC, Leist SR, Schafer A, Won J, Brown AJ, Montgomery SA, Hogg A, Babusis D, Clarke MO, Spahn JE, Bauer L, Sellers S, Porter D, Feng JY, Cihlar T, Jordan R, Denison MR, Baric RS.** 2020. Comparative therapeutic efficacy of remdesivir and combination lopinavir, ritonavir, and interferon beta against MERS-CoV. Nat Commun **11:**222.
- 7. **Goudreau N, Lemke CT, Faucher AM, Grand-Maitre C, Goulet S, Lacoste JE, Rancourt J, Malenfant E, Mercier JF, Titolo S, Mason SW.** 2013. Novel inhibitor binding site discovery on HIV-1 capsid N-terminal domain by NMR and X-ray crystallography. ACS Chem Biol **8:**1074-1082.
- 8. **Lemke CT, Titolo S, Goudreau N, Faucher AM, Mason SW, Bonneau P.** 2013. A novel inhibitor-binding site on the HIV-1 capsid N-terminal domain leads to improved crystallization via compound-mediated dimerization. Acta Crystallogr D Biol Crystallogr **69:**1115-1123.
- 9. **Merluzzi VJ, Hargrave KD, Labadia M, Grozinger K, Skoog M, Wu JC, Shih CK, Eckner K, Hattox S, Adams J, et al.** 1990. Inhibition of HIV-1 replication by a nonnucleoside reverse transcriptase inhibitor. Science **250:**1411-1413.
- 10. **Mitsuya H, Weinhold KJ, Furman PA, St Clair MH, Lehrman SN, Gallo RC, Bolognesi D, Barry DW, Broder S.** 1985. 3'-Azido-3'-deoxythymidine (BW A509U): an antiviral agent that inhibits the infectivity and cytopathic effect of human T-lymphotropic virus type III/lymphadenopathy-associated virus in vitro. Proc Natl Acad Sci U S A **82:**7096-7100.
- 11. **Yarchoan R, Klecker RW, Weinhold KJ, Markham PD, Lyerly HK, Durack DT, Gelmann E, Lehrman SN, Blum RM, Barry DW, et al.** 1986. Administration of 3'-azido-3'-deoxythymidine, an inhibitor of HTLV-III/LAV replication, to patients with AIDS or AIDS-related complex. Lancet **1:**575-580.
- 12. **Cahn P, Sued O.** 2007. Raltegravir: a new antiretroviral class for salvage therapy. Lancet **369:**1235-1236.
- 13. **Feng L, Sharma A, Slaughter A, Jena N, Koh Y, Shkriabai N, Larue RC, Patel PA, Mitsuya H, Kessl JJ, Engelman A, Fuchs JR, Kvaratskhelia M.** 2013. The A128T resistance mutation reveals aberrant protein multimerization as the primary mechanism of action of allosteric HIV-1 integrase inhibitors. J Biol Chem **288:**15813-15820.
- 14. **Wang H, Jurado KA, Wu X, Shun MC, Li X, Ferris AL, Smith SJ, Patel PA, Fuchs JR, Cherepanov P, Kvaratskhelia M, Hughes SH, Engelman A.** 2012. HRP2 determines the efficiency and specificity of HIV-1 integration in LEDGF/p75 knockout cells but does not contribute to the antiviral activity of a potent LEDGF/p75-binding site integrase inhibitor. Nucleic Acids Res **40:**11518-11530.
- 15. **Anderson J, Schiffer C, Lee SK, Swanstrom R.** 2009. Viral protease inhibitors. Handb Exp Pharmacol doi:10.1007/978-3-540-79086-0_4**:**85-110.
